# Pharmacokinetic herb-drug interactions between Aidi injection and doxorubicin in rats with diethylnitrosamine-induced hepatocellular carcinoma

**DOI:** 10.1186/s40360-021-00515-9

**Published:** 2021-09-06

**Authors:** Yuan Lu, Jie Pan, Xiaoqing Zhu, Shuai Zhang, Chunhua Liu, Jia Sun, Yueting Li, Siying Chen, Jing Huang, Chuang Cao, Yonglin Wang, Yongjun Li, Ting Liu

**Affiliations:** 1 State Key Laboratory of Functions and Applications of Medicinal Plants, Guizhou Provincial Key Laboratory of Pharmaceutics, Guizhou Medical University, Guiyang, 550004 China; 2 School of Pharmacy, Guizhou Medical University, No.9, Beijing Road, Yunyan District, Guiyang, 550004 China; 3 Engineering Research Center for the Development and Application of Ethnic Medicine and TCM (Ministry of Education), Guizhou Medical University, Guiyang, 550004 China; 4 Department of Interventional Radiology, The Affiliated Cancer Hospital of Guizhou Medical University, Guiyang, 550000 China

**Keywords:** Pharmacokinetic herb-drug interaction, Hepatocellular carcinoma, Aidi injection, Doxorubicin, Doxorubicinol, Rat

## Abstract

**Background:**

Aidi Injection (ADI), a Chinese herbal preparation with anti-cancer activity, is used for the treatment of hepatocellular carcinoma (HCC). Several clinical studies have shown that co-administration of ADI with doxorubicin (DOX) is associated with reduced toxicity of chemotherapy, enhanced clinical efficacy and improved quality of life for patients. However, limited information is available about the herb-drug interactions between ADI and DOX. The study aimed to investigate the pharmacokinetic mechanism of herb-drug interactions between ADI and DOX in a rat model of HCC.

**Methods:**

Experimental HCC was induced in rats by oral administration of diethylnitrosamine. The HCC rats were pretreated with ADI (10 mL/kg, intraperitoneal injection) for 14 consecutive days prior to administration of DOX (7 mg/kg, intravenous injection) to investigate pharmacokinetic interactions. Plasma concentrations of DOX and its major metabolite, doxorubicinol (DOXol), were determined using ultra-performance liquid chromatography-tandem mass spectrometry (UPLC-MS/MS).

**Results:**

Preadministration of ADI significantly altered the pharmacokinetics of DOX in HCC rats, leading to increased plasma concentrations of both DOX and DOXol. The area under the plasma drug concentration-time curve (AUCs) of DOX and DOXol in rats pretreated with ADI were 3.79-fold and 2.92-fold higher, respectively, than those in control rats that did not receive ADI.

**Conclusions:**

Increased levels of DOX and DOXol were found in the plasma of HCC rats pretreated with ADI.

## Background

Hepatocellular carcinoma (HCC) is one of the most common liver malignancies in regions where chronic hepatitis or liver diseases are prevalent, such as China [1]. Doxorubicin (DOX) is a key drug used in chemotherapy of HCC but its clinical utility is limited by both drug resistance and cardiotoxicity [2]. It has previously been suggested that the main active metabolite of DOX, doxorubicinol (DOXol), contributes to both the efficacy and toxicity of DOX [3].

HCC treatment is mainly inclined to comprehensive treatment. A large number of clinical trials have confirmed that natural products has a significant effect in the field of HCC, which not only improves the prognosis and quality of life of patients, but also improves the survival rate of patients [4–6]. Aidi Injection (ADI, Z52020236, China Food and Drug Administration), which contains extracts of *Astragali Radix*, *A. senticosus*, *Ginseng Radix* and *Mylabris* is widely used in China for the treatment of HCC. Several clinical studies have shown that combining ADI with chemotherapy reduces the toxicity of chemotherapy, enhances clinical efficacy and improves the quality of life of cancer patients [7–9]. Cantharidin, the major bioactive component of *Cantharis*, has potent antitumor activity, induces apoptosis in a variety of tumor cells, increases numbers of white blood cells and reduces the occurrence of bone marrow suppression [10–13]. Recent pharmacological studies have shown that *astragalus* polysaccharides have significant immunomodulatory activity [14], are hepatoprotective and antioxidant [15, 16], and have antitumor effects [17]. *A. senticosus* also has antitumor and immunomodulatory effects [18]. Ginsenosides, such as ginsenosides Rg_3_ and Rh_2_, show antitumor and antiangiogenic effects in various models using tumor cells and vascular endothelial cells [19, 20]. Many patients use ADI for HCC, before and after treatment with DOX, to reduce toxicity and improve the efficacy of chemotherapy [21, 22]. These effects are, however, insufficient to explain why ADI improves the clinical efficacy of chemotherapy drugs and the mechanism leading to increased efficacy needs to be explained. Plasma drug concentrations are generally believed to be proportional to the therapeutic effect and toxicity of a drug [23]. So far, there have been no reports describing research into pharmacokinetic herb-drug interactions between ADI and DOX and the nature of the interaction remains unknown.

There are potential risks in co-administering ADI with DOX in an outpatient setting and a pharmacokinetic study to evaluate potential interactions of ADI with DOX is needed. We hypothesized that ADI may alter the pharmacokinetics of DOX and used rats with experimental HCC to evaluate this hypothesis. HCC rats were pretreated with ADI (10 mL/kg, once a day, by intraperitoneal (*i.p.*) injection) for 14 consecutive days prior to administration of DOX (7 mg/kg by intravenous (*i.v.*) injection) to investigate pharmacokinetic interactions.

## Methods

### Chemicals and reagents

ADI (Product No. 20150627) was supplied by Guizhou Ebay Pharmaceutical Co., Ltd. (Guizhou, China). Diethylnitrosamine, doxorubicin (25316–40-9), doxorubicinol (54193–28-1), and the internal standard (IS) tropisetron hydrochloride (105826–92-4) were all purchased from Dalian Meilun Biotech Co., Ltd. (Liaoning, China). HPLC-grade acetonitrile, methanol and formic acid was supplied by Merck Company Inc. (Darmstadt, Germany). Distilled water was obtained from Watsons Group Co., Ltd. (Hong Kong, PRC). All chemicals and reagents used were of chromatographic or analytical grade.

### Animals

All experimental procedures were conducted according to the Institutional Animal Care guidelines and approved ethically by the Administration Committee of Experimental Animals, Guizhou Province, China. Male, pathogen-free, Sprague-Dawley rats (180–200 g) were purchased from Changsha Tianqing Biological Technology Co., Ltd. (Changsha, China, Certificate No. SCXK2016–0015) and acclimated for at least 1 week in their environmentally controlled quarters (25 °C ± 2 °C and 12/12 light/dark cycle), with free access to standard chow and water.

### Induction of HCC in rats by diethylnitrosamine

Experimental HCC was induced in rats by oral administration of diethylnitrosamine (DEN), as previously described [24, 25]. In brief, DEN (95 μg/mL) was administered in drinking water for 4 consecutive weeks, administration was interrupted for 4 weeks, and then resumed for 8 weeks.

### Animal treatment

On the last day of DEN administration, 24 HCC rats were randomly divided into two groups of 12 animals, a control group and an ADI group. The control group received saline (10 mL/kg, *i.p.*) once a day for 14 consecutive days and the ADI group received ADI (10 mL/kg, *i.p.*) once a day for 14 consecutive days. The rats were allowed free access to standard chow and water during these 14 days. Access to food was then prohibited for 12 h, with continued free access to water. Six rats from each group were then treated with DOX (7 mg/kg, *i.v.*). Blood samples (~ 250 μL) were collected from the tail vein into heparinized centrifuge tubes 0.033, 0.083, 0.167, 0.25, 0.333, 0.5, 1, 2, 4 and 8 h after DOX administration. Each blood sample was centrifuged for 5 min at 3306×*g* and an aliquot of the supernatant (100 μL) was transferred to a labeled plastic vial and stored at − 20 °C before analysis. At the end of study, all animals were euthanized by our veterinary staff in the animal care facility by carbon dioxide asphyxiation.

### Pharmacokinetic studies

#### UPLC-ESI-MS conditions

Chromatographic conditions were based on preliminary research work carried out in our laboratory [26]. A Waters ACQUITY UPLC system (Waters Corp., Milford, MA, USA), coupled with a Waters TQD Quantum triple-quadrupole mass spectrometer equipped with an electrospray ionization (ESI) source, was used for determination of the chromatographic analytes. Waters MassLynx software v.4.1 was used for acquisition and data processing. Separation and quantification were performed using a BEH C_18_ column (50 mm × 2.1 mm × 1.7 μm, Waters, Wexford, Ireland). The column temperature was 45 °C and the flow rate was 0.35 mL/min. The eluent was a mixture of mobile phase A (acetonitrile containing 0.1% formic acid) and mobile phase B (water containing 0.1% formic acid), with a gradient program as follows: 0–0.5 min, 10–30% A; 0.5–1.5 min, 30–60% A; 1.5–2.0 min, 60–90% A; 2.0–3.0 min, 90–10% A. The samples were kept at 25 °C in the sample manager. The injection volume was 1.0 μL (partial loop with needle overfill mode). A strong needle wash solution (90:10, methanol-water, *v/v*) and a weak needle wash solution (10:90, acetonitrile-water, *v/v*) were used. The mass spectrometer was operated in positive ion mode, with optimized parameters set as follows: nitrogen gas flow, 650 L/h; capillary voltage, 3 kV; ion source temperature, 120 °C; desolvation temperature, 350 °C. Cone voltages were optimized and set at 20 V, 20 V, and 32 V for DOX, DOXol, and IS, respectively. Quantification was performed using selected or single ion recording mode by monitoring the parent ions (m/z 544.3 for DOX, m/z 546.3 for DOXol and m/z 285.3 for IS).

#### Sample preparation

Samples were thawed to room temperature before analysis. IS solution [50 μL, 50 ng/mL IS dissolved in water/acetonitrile (50:60, *v/v*)] was added to rat plasma (100 μL). After vortexing for 5 min, methanol containing 5% formic acid (450 μL) was added to precipitate the proteins. After vortexing, mixing and sonication for 5 min, the sample was centrifuged at 13,000×*g* for 10 min. The supernatant was then transferred to another tube and evaporated to dryness under a gentle stream of nitrogen. The residue was dissolved in mobile phase (mobile phase A: mobile phase B, 10/90; 400 μL), centrifuged at 13,000×*g* for 10 min, and an aliquot (1 μL) of the solution was injected into the UPLC-MS/MS.

#### Pharmacokinetic analysis

Pharmacokinetic parameters were calculated using Drug and Statistic (DAS) pharmacokinetic software version 2.0 (Mathematical Pharmacology Professional Committee of China, Shanghai, China). All data were presented as the mean ± standard deviation (SD). A two tailed Student’s *t*-test was used to determine the significance of differences in pharmacokinetic parameters between the control group and the ADI group. *P* < 0.05 was considered to be statistically significant.

## Results

### Method validation

**Fig. 1:**
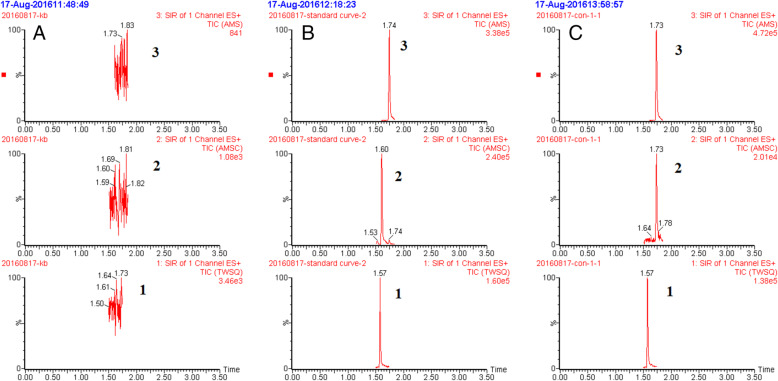
Chromatograms of DOX, DOXol and tropisetron (I.S.) in rat plasma. Blank plasma sample (A); a plasma sample spiked with DOX, DOXol, and tropisetron (I.S.), respectively (B); and a plasma sample obtained from a rat 10 min after intravenous administration of doxorubicin (C). 1.Tropisetron 2. Doxorubicinol 3. Doxorubicin

Plasma concentrations of DOX and DOXol were quantified using a validated UPLC-MS method previously developed in our laboratory [26]. Briefly, the retention times of tropisetron (IS), DOXol and DOX were 1.57, 1.60 and 1.74 min, respectively. Chromatograms of DOX, DOXol and IS in rat plasma is shown in Fig. 1. The lower limits of quantification (LLOQ) were 100 ng/mL for DOX and 10 ng/mL for DOXol. The mean recoveries of DOX and DOXol were 83.25–96.58% and the intra- and inter-day precisions were < 10%. DOX and DOXol in the analytical samples were stable for 12 h in the autosampler, for 72 h at − 20 °C and over three freeze-thaw cycles. Linearity, sensitivity, selectivity, accuracy, intra- and inter-day precision and stability of the method were validated according to the requirements for bioanalytical methods laid out in the Guidance for Industry Bioanalytical Method Validation Document from the American Food and Drug Administration.

### Pharmacokinetic study

**Fig. 2:**
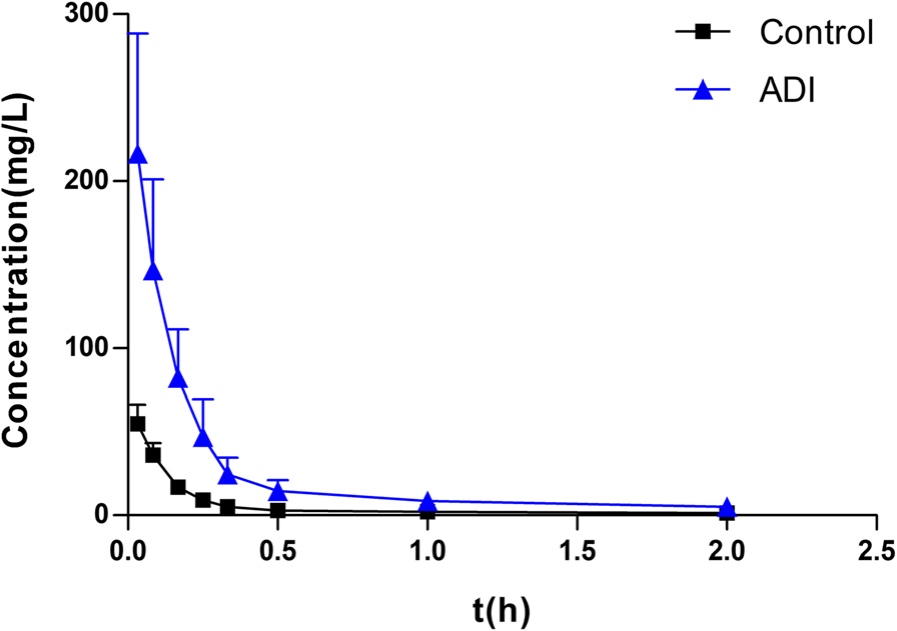
Plasma concentration-time profiles (Mean ± SD, *n* = 6) of DOX in control and ADI groups

**Fig. 3:**
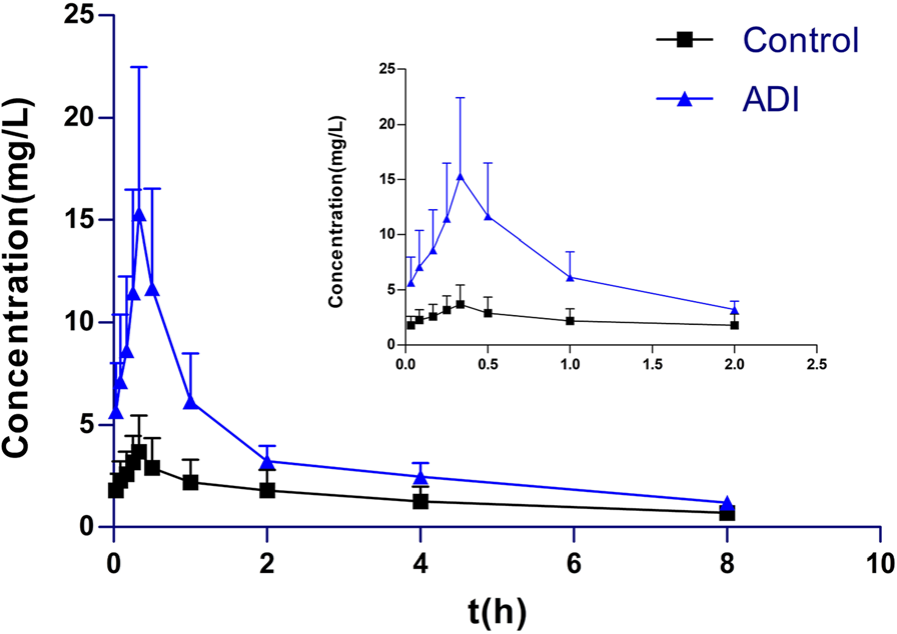
Plasma concentration-time profiles (Mean ± SD, *n* = 6) of DOXol in control and ADI groups

The effects of ADI on the pharmacokinetics of DOX in HCC rats were examined by administering a single dose of DOX (7 mg/kg, *p.o.*) to the rats. Pharmacokinetic investigation showed that the plasma concentration-time data for DOX were best fitted to a two-compartment intravenous open model. A one-compartment model was used to describe the pharmacokinetics of DOXol. Plasma concentrations of DOX were found to be significantly higher in the ADI group than in the control group (Fig. 2 and Table 1). The area under the plasma drug concentration-time curve (AUC) of DOX in the ADI group was 3.79-fold higher than that in the control group (*P* < 0.01). The half-life of distribution (t_1/2α_), apparent volume of distribution in the central compartment (V_1_) in the ADI group were also significantly higher than in the control group (*P* < 0.01). Meanwhile, compared with the control group, clearance (CL) of DOX in the ADI group was significantly lower (*P* < 0.05). There were no statistically significant differences in elimination half-life (t_1/2β_), elimination rate constant of drug from compartment 1 (K_10_), rate constant for movement of drug from compartment 1 to compartment 2 (K_12_) or rate constant for movement of drug from compartment 2 to compartment 1 (K_21_) between the two groups (*P* > 0.05). The AUC of DOXol, the main metabolite of DOX, was 2.92-fold higher in the ADI group than in the control group (Fig. 3 and Table 2). The mean residence time (MRT_0-t_), elimination half-life time (t_1/2z_) and peak concentration (C_max_) of DOXol were all significantly increased in the ADI group compared with the control group (2.22-, 2.39- and 3.46-fold, respectively) (*P* < 0.05). Pharmacokinetic analysis thus showed that preadministration of ADI significantly altered the pharmacokinetics of DOX in HCC rats, leading to elevation of plasma concentrations of both DOX and DOXol.

**Table 1:** Pharmacokinetic parameters of DOX in control and ADI groups after single intravenous administration of DOX (7 mg/kg) ($\overline{X}$ ± SD, *n* = 6)

Parameters	Control	ADI
t_1/2α_ (h)	0.074 ± 0.005	0.089 ± 0.017**
t_1/2β_ (h)	2.734 ± 1.385	2.638 ± 1.108
V_1_ (L/kg)	0.099 ± 0.026	0.028 ± 0.011**
CL (L/h/kg)	0.37 ± 0.15	0.099 ± 0.048*
AUC_0-t_ (mg/L*h)	20.21 ± 11.22	76.50 ± 34.89**
K_10_ (1/h)	3.91 ± 1.67	3.68 ± 1.69
K_12_ (1/h)	5.26 ± 2.05	4.27 ± 0.73
K_21_ (1/h)	0.77 ± 0.49	0.65 ± 0.48

Data are presented as mean ± SD (*n* = 6). *P < 0.05, **P < 0.01 versus control group

t_½α_, half-life of distribution; t_½β_, half-life of elimination; V_1_, apparent volume of distribution in central compartment; CL, clearance; AUC_0-t_, area under plasma drug concentration-time curve; K_10_, elimination rate constant of drug from compartment 1; K_12_, rate constant for movement of drug from compartment 1 to compartment 2; K_21_, rate constant for movement of drug from compartment 2 to compartment 1

**Table 2:** Pharmacokinetic parameters of DOXol in control and ADI groups after single intravenous administration of DOX (7 mg/kg) ($\overline{X}$ ± SD, *n* = 6)

Parameters	Control	ADI
AUC_0-t_ (mg/L*h)	5.62 ± 2.23	16.41 ± 2.91**
MRT_0-t_ (h)	2.40 ± 0.32	5.33 ± 1.31**
t_1/2z_ (h)	2.09 ± 0.98	4.99 ± 0.95**
T_max_ (h)	0.33 ± 0.001	0.33 ± 0.001
CLz/F (L/h/kg)	1.33 ± 0.57	0.43 ± 0.08
Vz/F (L/kg)	4.43 ± 3.86	3.13 ± 0.98
C_max_ (mg/L)	2.41 ± 1.04	8.34 ± 3.90**

Data are presented as mean ± SD (*n* = 6). **P < 0.01 versus control group

AUC_0-t_, area under plasma drug concentration-time curve; MRT_0-t_, mean residence time; t_1/2z_, half-life of elimination; T_max_, peak time; CLz, clearance; Vz, apparent volume of distribution; C_max_, maximum (peak) plasma drug concentration

## Discussion

Despite improved diagnostic tools for HCC and much better survival rates of patients, the outcomes and prognoses of HCC patients remain poor because of poor liver function and advanced cancer stage. Transcatheter arterial chemoembolization (TACE) is the main treatment for unresectable HCC, and DOX is one of the commonly used drugs in TACE [27, 28]. TACE is not, however, ideal as a long-term cure since it often reduces immunity, aggravates the impairment of liver function and reduces life quality of HCC patients [29]. Finding a way to reduce liver injury and improve clinical efficacy and quality of life has thus become a key issue and many patients in Asia are seeking help from traditional herbal medicines [30, 31].

ADI(Z52020236) is the exclusive product of Guizhou Ebay Pharmaceutical Co. LTD, which was approved for clinical use since 2002. ADI, combined with TACE, is now widely used in the treatment of unresectable HCC in China. It has been reported that this combination can, to some extent, enhance the clinical effect, improve overall survival, increase quality of life for patients and reduce adverse events, including leukopenia, gastrointestinal side effects and liver damage [32]. In a previous study, we found that ADI reduced serum levels of alanine aminotransferase, aspartate aminotransferase, total bilirubinand alkaline phosphatasein rats with DEN-induced HCC, confirming its protective effect on liver function [26]. The clinical use of ADI is intravenous drip, 50 to 100 ml of ADI for adults, mixed with 0.9% sodium chloride injection or glucose injection, once a day. When combined with radiotherapy and chemotherapy, the course of treatment is synchronized with radiotherapy and chemotherapy. ADI is used for 10 days before and after surgery. For patients with advanced cachexia, ADI is used for 30 days or depending on the condition. According to human and rat dose conversion and the convenience of practical operation, rats were injected intraperitoneally with 10 mL/kg of ADI for 14 consecutive days [33].

Animal models of HCC are essential for understanding the cellular and molecular aspects of tumor development, as well as pharmacological and pharmacokinetic testing. Although the genetic engineering model of HCC can be used to prove the carcinogenic and tumor suppressive effects of cell and viral genes [34], chemically induced HCC models can cause both cirrhosis and HCC. The DEN-induced liver cancer model produces HCC in the context of fibrosis, similar to the liver microenvironment of almost all HCC patients [35]. Therefore, this method was adopted for experiments. The pharmacokinetic interaction between ADI and DOX was investigated in the 14th day after the end of DEN administration, we believe that DEN should disappear in the body, as the time between DEN and ADI is long enough. The main limitation of this experiment is that only one liver cancer model is used. To eliminate DEN interference, in the future, we will use other models to verify the results of this experiment, such as transplanted tumor models or spontaneous liver cancer models. In addition, we will investigate the distribution of DOX and DOXol in the tumor tissue when DOX is co-administered with ADI in subsequent experiments.

Our pharmacokinetic results show that plasma concentrations of DOX and DOXol were significantly higher in the ADI group. AUC of DOX and DOXol in the ADI group was 3.79-fold and 2.92-fold higher than that in the control group (both *P* < 0.01). It means there is herb-drug interactions between ADI and DOX. ADI can change the pharmacokinetics of DOX in HCC rats. To the best of our knowledge, the plasma protein binding rate of DOX is very low. Therefore, ADI is unlikely to change the plasma concentration of DOX and DOXol by competing with plasma protein binding. ADI alters DOX’s drug metabolism enzymes and transporters is a possible cause. DOX is mainly metabolized in the liver and excreted in bile, 50% of which are parent drugs, and 23% are active metabolites such as DOXol [36]. DOX can be converted into a semiquinone structure through single-electron reduction, and it can also form DOXol through C-13 hydroxylation in the cytoplasm by carbonyl reductase 1 (CBR1), which generally expressed in liver, heart and other tissues [37]. Various transporters, particularly P-gp (ABCB1, MDR1) and ABCC1 (MRP1), are thought to be play a role in resistance to DOX [2]. Generally, increased expression of P-gp results in increased DOX efflux. Many studies have shown that the resistance of DOX can be overcome by inhibiting P-gp [38–40]. The import transporter SLC22A16 has also been shown to be involved in intracellular transport of DOX [41]. Therefore, if ADI changes the activity or expression of CBR1, SLC22A16 and P-gp, it would change the pharmacokinetic behavior of DOX.

In our previous study, ADI also reduced mRNA levels and enzymatic activity of glutathione transferases (GSTs), and decreased protein expression of GST-π in the livers of HCC rats [24]. High expression of GST-π is known to accelerate the transformation and metabolism of anti-tumor drugs, shorten the duration of effective drug concentrations in cells and rapidly reduce the accumulation of drugs in target sites, thus reducing efficacy [42]. GSTs are considered to be potential targets to overcome chemoresistance in solid tumors [43], and reduction of GSTs activity may be the one of underlying mechanisms for the synergistic effect of ADI. Inhibition of GSTs activity cannot, however, explain why administration of ADI leads to elevated levels of DOX and DOXol, since GSTs are not involved in DOX metabolism. In summary, to explain why ADI changed pharmacokinetics of DOX, more experiments with rigorous design are needed.

DOX is an effective chemotherapeutic drug. DOXol is the most important component of DOX-induced cardiotoxicity. Hence, increased blood concentrations of DOX and DOXol, in addition to implying that it may increase the therapeutic effect of DOX and may lead to stronger toxic and side effects. However, many studies have shown that the related ingredients of *Astragalus*, *A. senticosus*, *Ginseng* can play a synergistic effect, protect the heart, and reduce the toxic and side effects of chemotherapy drugs, such as ginsenoside Rg1 [44], ginsenoside Rg3 [45–47], astragalus polysaccharide [48, 49], acanthopanax senticosides B [50]. Therefore, how the combination of ADI and DOX can enhance the efficacy of DOX and reduce its myocardial toxicity requires more experiments to verify.

## Conclusions

In this study, an accurate and validated UPLC-MS/MS method was developed to determine DOX and DOXol concentrations in rat plasma and then used to investigate DOX pharmacokinetics. Using this method, we identified potential herb-drug interactions between ADI and DOX. The AUCs of DOX and DOXol in rats pretreated with ADI were 3.79-fold and 2.92-fold higher, respectively, than AUCs in the control group. Further studies are needed to better understand the synergistic effect of ADI and DOX. Since both DOX and DOXol are implicated in the cardiotoxicity, in future studies we will investigate the cardiotoxicity of DOX and the distribution of DOX and DOXol in the heart and tumor tissue when DOX is co-administered with ADI.

## Data Availability

The datasets used and/or analyzed during the current study are available from the corresponding author on reasonable request.

## Abbreviations

ADI: Aidi Injection
DOX: Doxorubicin
DOXol: Doxorubicinol
UPLC-MS/MS: Ultra-performance Liquid Chromatography-Tandem Mass Spectrometry
DEN: Diethylnitrosamine
IS: Internal standard
AUC: The area under the plasma drug concentration-time curve
t_1/2α_: The half-life of distribution
V_1_: The apparent volume of distribution in the central compartment
CL: The clearance
t_1/2β_: The elimination half-life
K_10_: The elimination rate constant of drug from compartment 1
K_12_: The rate constant for movement of drug from compartment 1 to compartment 2
K_21_: The rate constant for movement of drug from compartment 2 to compartment 1
MRT_0-t_: The mean residence time
t_1/2z_: The elimination half-life time
C_max_: The peak concentration
